# The Combination of Immune Checkpoint Blockade and Angiogenesis Inhibitors in the Treatment of Advanced Non-Small Cell Lung Cancer

**DOI:** 10.3389/fimmu.2021.689132

**Published:** 2021-06-02

**Authors:** Sijia Ren, Xinxin Xiong, Hua You, Jianfei Shen, Penghui Zhou

**Affiliations:** ^1^ Taizhou Hospital, Zhejiang University School of Medicine, Taizhou, China; ^2^ Guangdong Provincial People’s Hospital, Guangdong Academy of Medical Sciences, Guangzhou, China; ^3^ Medical Oncology Department, Affiliated Cancer Hospital & Institute of Guangzhou Medical University, Guangzhou, China; ^4^ State Key Laboratory of Oncology in Southern China, Collaborative Innovation Center for Cancer Medicine, Sun Yat-sen University Cancer Center, Guangzhou, China

**Keywords:** NSCLC, immunotherapy, immune checkpoint blockade, angiogenesis inhibitors, combination therapy, tumor microenvironment

## Abstract

Immune checkpoint blockade (ICB) has become a standard treatment for non-small cell lung cancer (NSCLC). However, most patients with NSCLC do not benefit from these treatments. Abnormal vasculature is a hallmark of solid tumors and is involved in tumor immune escape. These abnormalities stem from the increase in the expression of pro-angiogenic factors, which is involved in the regulation of the function and migration of immune cells. Anti-angiogenic agents can normalize blood vessels, and thus transforming the tumor microenvironment from immunosuppressive to immune-supportive by increasing the infiltration and activation of immune cells. Therefore, the combination of immunotherapy with anti-angiogenesis is a promising strategy for cancer treatment. Here, we outline the current understanding of the mechanisms of vascular endothelial growth factor/vascular endothelial growth factor receptor (VEGF/VEGFR) signaling in tumor immune escape and progression, and summarize the preclinical studies and current clinical data of the combination of ICB and anti-angiogenic drugs in the treatment of advanced NSCLC.

## Introduction

Lung cancer is one of the most common cancer types with high mortality in the world (1). Adenocarcinoma, squamous cell carcinoma and large cell carcinoma are the three major kinds of NSCLC comprising 85% of all lung cancers (2). Because of the lack of early diagnosis indicators, more than 70% of cancer patients have experienced local invasion, lymph node and distant metastasis at the first diagnosis (3). These patients have extremely poor prognoses. The five-year survival rate of patients at this stage is only 4% (4).

In the past decade, immunotherapy has made significant progress for the treatment of NSCLC. Improving the therapeutic effect *via* combination strategy has become the main direction in the field. A number of clinical trials testing the combination of immunotherapy and anti-angiogenesis have shown promising results in different tumor types including NSCLC. However, due to the complicated regulatory mechanisms of these two kinds of therapies, how to collaboratively use them to obtain the maximal therapeutic effect remains to be answered. Understanding the potential mechanisms of combination might help to select appropriate patients and treat them at right timing with optimized dosages of drugs.

## Immune Checkpoints and Inhibitors

Immune checkpoint inhibitors (ICIs) are widely used in the treatment of NSCLC. A series of receptor/ligand pairs such as CD28-CTLA4/B7 and programmed cell death-1/programmed death ligand 1 (PD-1/PD-L1) are involved in the antitumor immune response at different stages (5, 6). These costimulatory and coinhibitory receptor/ligand pairs are collectively referred to as immune checkpoints (7). PD-1 is expressed on a variety of immune cells, such as T cells, NK cells, B cells, and monocytes (8). The PD-1 pathway mediates inhibitory signaling triggered by the binding to PD-L1. PD-L1 expressed on cancer cells could suppress effector T cells and thus prevent T cell-mediated tumor destruction (9). Therefore, blocking the PD-1/PD-L1 inhibitory pathway can reactivate the immune attack on tumor cells, thereby treating cancer (10).

A number of PD-1, PD-L1 and CTLA-4 inhibitors, including Pembrolizumab (11), nivolumab (12), atezolizumab (13), durvalumab (14), avelumab (15) and ipilimumab (16), have been approved for the treatment of advanced NSCLC. Pembrolizumab and nivolumab have been approved by the U.S. Food and Drug Administration (FDA) for the treatment of non-small cell lung cancer with positive PD-L1 expression. The PACIFIC (17) Phase III clinical trial (NCT02125461) in Europe makes durvalumab the only phase III immunotherapy drug recommended by the current guidelines. Japan is also conducting trails of atezolizumab, such as J-TAIL (NCT03645330) (https://clinicaltrials.gov/ct2/show/NCT03645330), J-TAIL-2 (NCT04501497) (https://clinicaltrials.gov/ct2/show/NCT04501497), and durvalumab, AYAME (NCT03995875) (https://clinicaltrials.gov/ct2/show/NCT03995875). In China, according to the ORIENT-11 study (NCT03607539), sintilimab has been approved as the first-line treatment for non-squamous NSCLC combined with pemetrexed and platinum chemotherapy. The Phase III trial (NCT03134872) (18) of SHR-1210 combined with pemetrexed and carboplatin in the treatment of non-squamous non-small cell lung cancer is also ongoing. Nevertheless, due to the tumor heterogeneity and the complexity of the tumor microenvironment (TME), the overall response rates to ICI therapy keep at low levels (19). To increase the therapeutic efficacy, combination strategies have become the major focus of cancer immunotherapy (20). A large number of clinical trials are testing the combination of immunotherapy with traditional therapies such as surgery, chemotherapy, radiotherapy, targeted therapy and other treatment methods.

ICIs obtain therapeutic effect by inducing a durable antitumor immune response (21). However, high levels of immunosuppressive cells in the TME and insufficient infiltration of effector cells into tumor severely impair the antitumor immunity, and thus decreasing the efficacy of ICIs. Recent studies have shown that pro-angiogenic factors in tumor promote the development of immunosuppressive cells, and neovessels reduce the infiltration of effector cells (22). The combination with anti-angiogenic agents is thought to be a promising strategy to enhance the therapeutic efficacy of ICIs.

## Tumor Angiogenesis and Inhibitors

**Figure 1:**
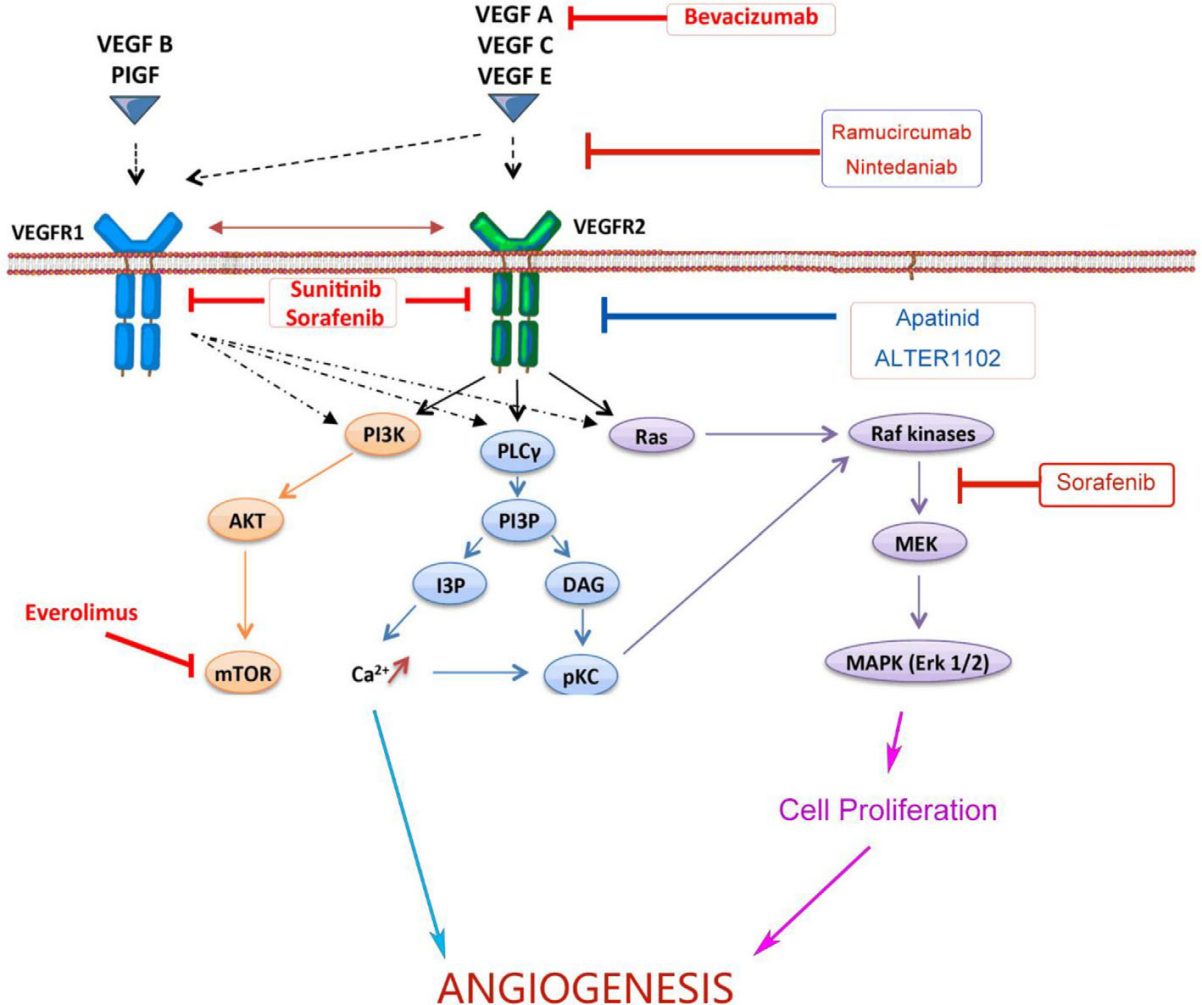
Monoclonal antibodies and small molecules targeting VEGF/VEGFR signaling in tumor angiogenesis. Monoclonal antibodies and small molecule TKIs targeting the VEGFA/VEGFR-2/PLCγ/Raf/PI3K signaling pathway could inhibit tumor angiogenesis and improve the efficiency of anticancer treatments. VEGF, Vascular Endothelial Growth Factor; VEGFR, Vascular Endothelial Growth Factor Receptor; TKI, Tyrosine Kinase Inhibitor; PI3K, Phosphoitide 3-Kinase; AKT, serine/threonine-specific protein kinase; mTOR, mammalian target of rapamycin; PLCγ, Phospholipase C γ; PI3P, Phosphatidylinositol 3-Phosphate; IP3, Inositol Triphosphate; DAG, Diacyl Glycerol; pKC, Protein Kinase C; MEK, Mitogen-activated protein kinase; MAPK, Mitogen Activated Protein Kinase.

Angiogenesis is a hallmark of cancer associated with occurrence, proliferation and metastasis of tumors (23). Targeting the angiogenesis pathway has been found to be effective in the treatment of a variety of cancers including NSCLC. The abnormal structure and function of tumor angiogenesis facilitate the development of a hostile tumor microenvironment characterized by increased interstitial pressure, hypoxia and acidosis (24). Hypoxia further induces the expression of genes involved in blood vessel formation and cell proliferation, and thus exacerbating the TME (25). VEGFs, a family of secreted glycoproteins, play an essential role in the angiogenesis of tumor, which include VEGF-A, VEGF-B, VEGF-C, VEGF-D, VEGF-E, VEGF-F, placental growth factor (PIGF) (26). There are three VEGF receptors, VEGFR-1, -2 and -3. The effect of VEGF in promoting angiogenesis is mainly mediated by VEGFR-2. Signaling pathways downstream VEGFR-2, such as phospholipase C gamma (PLCγ), Raf and phosphoinositide-3-kinase (PI3K) (22), promote angiogenesis and vascular permeability by regulating the differentiation, migration, proliferation and survival of microvascular endothelial cells (27). Both monoclonal antibodies blocking the interaction between VEGF and VEGFR or small molecules targeting downstream signaling could inhibit tumor angiogenesis (28). As listed in **Figure 1**, both monoclonal antibodies and small molecule inhibitors interfering angiogenesis have been approved for the treatment in various cancer types.

Bevacizumab, or Avastin, is a humanized monoclonal antibody binding to VEGF-A. It has been approved for the treatment of advanced non-squamous NSCLC. Phase III clinical trials showed that bevacizumab combined with carboplatin and paclitaxel significantly improved the therapeutic efficacy (29). Ramucirumab is a recombinant human IgG1 monoclonal antibody targeting VEGFR2. According to the results of the REVEL study, the FDA and European Medicines Agency (EMA) have approved the combination of Ramucirumab and docetaxel for the treatment of metastatic NSCLC and progressed disease after the treatment of platinum (30).

Nintedanib is a small molecular inhibitor targeting three critical receptors signaling in angiogenesis, VEGFR, fibroblast growth factor receptor (FGFR) and platelet-derived growth factor receptor (PDGFR). The LUME-Lung 1 study showed that nintedanib in combination with pemetrexed significantly improved progress-free survival (PFS) of patients (31). It was approved by EMA as the second-line treatment for stage IV NSCLC. In addition, tyrosine kinase inhibitors (TKIs) including sorafenib, sunitinib and apatinib have also been clinically studied in advanced NSCLC, but no obvious overall survival (OS) benefit was observed. Anlotinib is another small molecular inhibitor targeting multiple receptor tyrosine kinases (RTKs), including VEGFR2 and VEGFR3. The results of the ALTER 0303 trial showed that anlotinib significantly prolonged the OS and PFS of patients with advanced NSCLC (32). It has been approved as the third-line treatment for advanced NSCLC.

Although a number of angiogenesis inhibitors have been tested in clinical trials, anti-angiogenesis alone showed limited therapeutic effect in cancer treatment (33). Most of the angiogenesis inhibitors were approved for the combination therapy with other drugs. Given that reduced vessels in tumor will result in decreased delivery of combinatory drugs as well, these results challenge the well-accepted mechanism of anti-angiogenesis in reducing vascular supply, and thus suppress tumor growth by starving tumor. This paradox is resolved by recent findings of vessel normalization, a process recovering the perfusion function and structure of vessels in tumor, which enhanced antitumor immune response by increasing immune cell infiltration and oxygen supply in tumor (33–36). Consistent with the mechanism of vessel normalization, low dose of anti-VEGFR2 antibody showed better effect on reprogramming the tumor microenvironment and displayed better therapeutic efficacy than the high-dose treatment (37). The vessel normalization theory provides novel perspectives in the combination of anti-angiogenesis with other drugs or therapies.

## Rationale for Combination of ICI Inhibitors With Angiogenesis in NSCLC

### Angiogenesis Fosters An Immunosuppressive Tumor Microenvironment by Modifying The Recruitment of Immune Cells

TME is a dynamic ecosystem composed of tumor cells, immune cells, fibroblasts, stroma cells, blood vessels and various soluble factors, which suppress antitumor immune response and promote resistance to immunotherapy (38). Excessive VEGF signaling drives aberrant angiogenesis in tumor. Compared to normal blood vessels in tissues, blood vessels in TME are leaky, tortuous, cystic dilation, interlaced and randomly connected. The tumor vascular endothelial cells have abnormal morphology, loose connections between pericytes and varied basement membrane thickness. These abnormalities of structure and function lead to the heterogeneity of tumor blood perfusion, and eventually form a microenvironment characterized by increased interstitial fluid pressure, hypoxia and acidosis (39). The hypoxic microenvironment induced by VEGF/VEGFR signaling suppresses the antitumor immune response through a variety of mechanisms (40, 41).

The TME is enriched with suppressive immune cells including regulatory T cells (Tregs), myeloid-derived suppressive cells (MDSCs), tumor associated macrophages (TAMs), and immature dendritic cells (imDC). Hypoxia facilitates the infiltration of these suppressive immune cells by inducing the expression of chemokines recruiting these immune cells. For example, C-C motif chemokine ligand 22 (CCL22) and C-C motif chemokine ligand 28 (CCL28) recruits Tregs into tumor (42); colony Stimulating factor 1 (CSF1), C-C motif chemokine ligand 2 (CCL2) and C-X-C motif chemokine ligand 12 (CXCL12) increases the recruitment of pro-inflammatory monocytes and TAMs, and convert TAMs from a pro-inflammatory M1-like type to a tumor-promoting M2-like type (43); Dendritic cells (DCs) are mainly recruited into tumor by C-C motif chemokine ligand 20 (CCL20), and granulocyte-macrophage colony stimulating factor (GM-CSF), Interleukin-6 (IL-6), Interleukin-10 (IL-10) prevent maturation of recruited DCs (44). Moreover, the hypoxic environment inhibits the infiltration of effector T cells. VEGF can reduce the expression of adhesion molecules critical for T cell infiltration, such as integrin ligand vascular cell adhesion protein 1 (VCAM1) and intercellular adhesion molecule 1 (ICAM1), on immune cells and endothelial cells (ECs) (45). VEGF-A, IL-10 and prostaglandin E2 (PGE2) induce the expression of Fas ligand on endothelial cells, which causes cell death of endothelial cells and CD8^+^ T cells through the Fas/FasL signaling pathway, and thus reduce T cell mobilization and infiltration (46). Consistently, blockade of the VEGF signaling reduced the recruitment of suppressive cells into tumor but increased the infiltration of effector T cells (37), indicating that anti-angiogenesis is a potential strategy to re-program the immunosuppressive TME, and thus improve the efficacy of immunotherapy.

### Angiogenic Factors Directly Regulate Differentiation of Various Immune Cells

**Figure 2:**
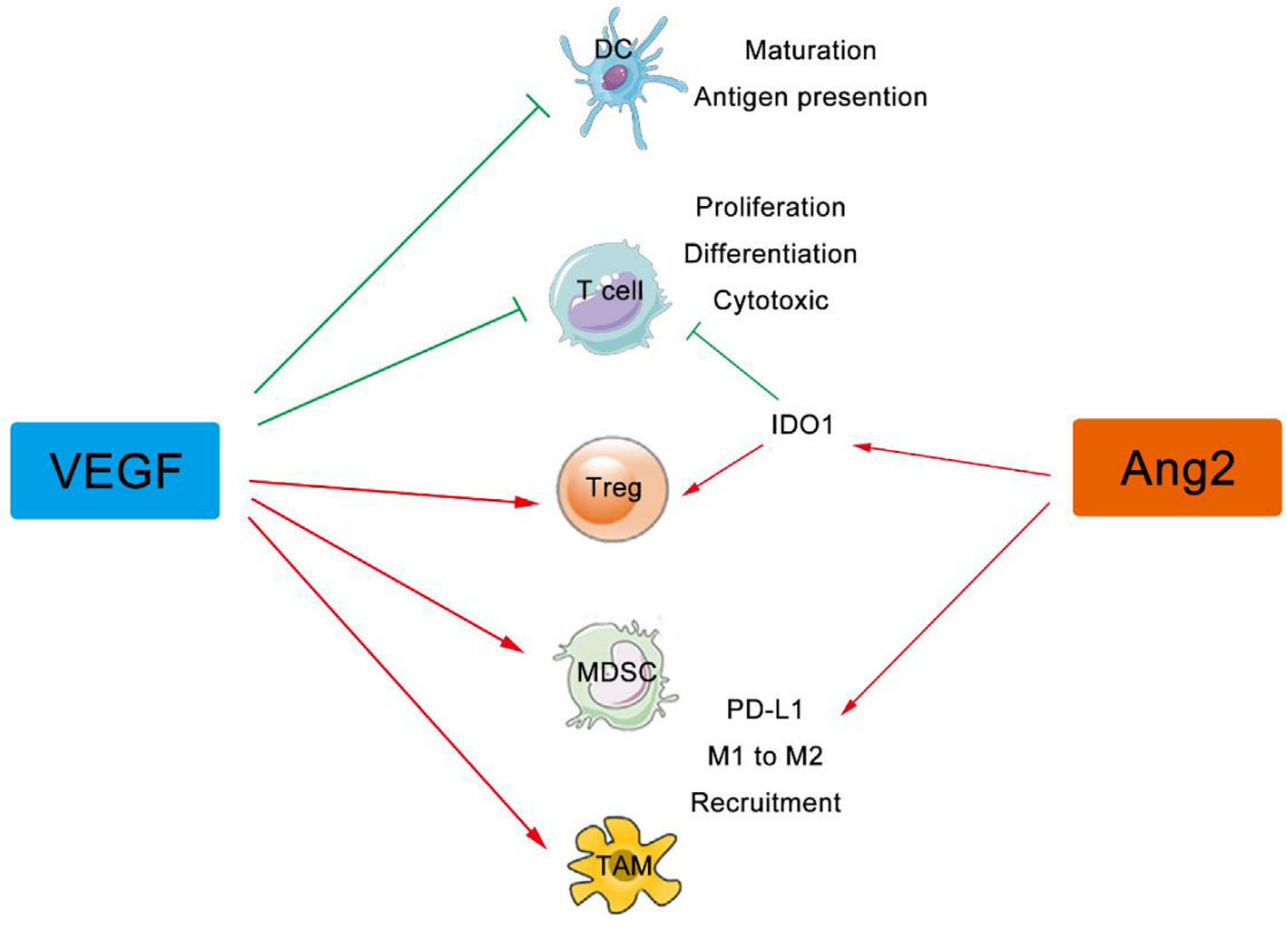
VEGF and ANG2 regulate immune cells in tumor. The VEGF family can suppress the maturation, differentiation, and antigen presentation of APCs, DCs, NKs, and T cells, while both VEGF and Ang2 can improve the suppressive effect of Tregs, TAMs, and MDSCs. VEGF, Vascular Endothelial Growth Factor; ANG2, Angiogenin 2; APCs, Antigen Presenting Cells, DCs, Dendritic Cells; Treg, Regulatory T cells; NKs, Natural Killer Cells; TAMs, Tumor Associated Macrophages; MDSCs, Myeloid Derived Suppressor Cells.

In addition to its effect on immune cell migration, the VEGF signaling directly regulates differentiation and proliferation of suppressive immune cells including Tregs, TAMs, MDSCs, and DCs (47, 48). VEGF (red stars) and angiopoietin-2 (ANG2) (green pentagons) are also produced by these immune cells, which foster both the paracrine and the autocrine VEGF (and/or ANG2) signaling in tumor (49). Immunosuppressive cytokines secreted by these suppressive immune cells, including IL-10, indoleamine 2,3-dioxygenase (IDO), and transforming growth factor beta (TGF-β) et al., further worsen the environment by inducing Tregs and inhibiting DC maturation, NK cell activation, T cell activation and proliferation (50). Therefore, angiogenesis inhibitors might normalize the aberrant vasculature in tumor, reduce the development of suppressive immune cells, enhance effector cell infiltration into tumor, and thus reprogram the immunosuppressive to immunosupportive (**Figure 2**).

#### VEGF Inhibits the Maturation and Differentiation of DCs

DCs are the professional antigen-presenting cells (APCs) which play a critical role in the antitumor immune cycle. Following the exposure to tumor antigens, DCs migrate to lymph nodes and become mature during the migration. They initiate adaptive antitumor immune response by activating T cells recognizing tumor antigens (51). Plenty of evidence has shown that VEGF could inhibit differentiation and maturation of DCs (52, 53). It was found that elevated VEGF levels in mice hindered the development of DCs (48). Studies have showed that VEGF-A inhibited the differentiation of monocytes to DC, and VEGF-A inhibition using bevacizumab or sorafenib restored this process (54).

Due to the lack of costimulatory molecules, immature DCs promote tolerance instead of activation of T cells. It was reported that the binding of VEGF to VEGFR-2 on the surface of DC restrains its maturation by inhibiting the nuclear factor κB (NF-κB) signaling pathway (55). VEGF inhibition increases antigen uptake and migration of tumor-associated DCs in mouse tumor models (56). The VEGFR inhibitor Axitinib promotes maturation of monocyte-derived human DCs, featured with elevated levels of activation markers, major histocompatibility complex (MHC) molecules and co-stimulatory genes such as CD80, CD86, and CD83 (57).

#### VEGF Increases the Number of Tregs

It is known that Tregs in tumor suppress T cell response against cancer (58). Studies have shown that the VEGF signaling contributes to the induction, maintenance and activation of Tregs in tumors. The expression of VEGF was found to be positively associated with the levels of Tregs in tumor, which indicate poor prognosis in many cancer types (59). Consistent with this finding, higher expression of VEGFR2 was found in Tregs compared to other CD4^+^ T cells (59, 60), suggesting a preferential role of VEGF signaling in Tregs. Interestingly, neuropilin-1, an co-receptor increasing the binding affinity of VEGF for VEGFRs, is also highly expressed in Tregs (61), which mediates the activation of Tregs and thus enhances their suppressive function (62). VEGF can directly bind to Neuropilin 1 (Nrp-1) on Tregs and guide their migration into a tumor (63). Inhibition of VEGF signaling using sunitinib, bevacizumab or soluble VEGFR-1/-2 reduce Treg proportion in different mouse tumor models and in cancer patients (47, 64–66). Decreased proliferation of Tregs and reduced levels of peripheral Treg levels are also reported in some studies. Following the reduction of Tregs, enhanced antitumor immune response was detected in tumors.

#### VEGF Promotes the Expansion of MDSCs

MDSCs were initially defined as CD11b^+^Gr-1^+^ cells in tumors. There are two main major populations of MDSCs: monocytic MDSCs (M-MDSC) and polymorphonuclear MDSCs (PMN-MDSC). PMN-MDSCs are the dominant population of MDSCs in mouse tumor models, while M-MDSCs are mainly found in human tumors (67). MDSCs employ a number of mechanisms to suppress the antitumor immune response, for examples, consuming the nutrient of lymphocyte, reducing trafficking and viability of lymphocyte, generating oxidative stress, and inducing the differentiation of Tregs (67, 68).

The intratumoral level of MDSCs was found to be associated with the VEGF concentration in mouse tumor models. In addition, VEGF infusion significantly elevated levels of Gr1^+^ cells in normal mice without tumor (48), suggesting that VEGF signaling is involved the differentiation of myeloid cells. It was reported that VEGF-A-induced excessive activation of Janus kinase 2/Signal transducer and activator of transcription 3 (Jak2/STAT3) signaling contributes to the abnormal myeloid cell differentiation in cancer (69). Inhibition of VEGF signaling by sunitinib decreased the levels of MDSC in the spleen, bone marrow, and tumor in mouse models, and showed combinatory effect with HPV vaccine for the treatment of tumors expressing human papillomavirus (HPV) antigens (70). Mechanistically, sunitinib downregulates STAT3 signaling and leads to apoptosis in MDSCs (71). In addition to the reduction in MDSC quantity, VEGF inhibition impairs their suppressive function. Axitinib treatment decreases the suppressive capacity of MDSCs isolated from spleens or tumors in mouse models. Moreover, axitinib promotes the differentiation of MDSC toward a phenotype with enhanced capacity of antigen presentation (72). Reduction of MDSCs was also observed in cancer patient treated with sunitinib, which led to stronger T cell immune response against cancer (73). A recent study also showed that bevacizumab-containing regimens had low levels of the granulocytic MDSCs than regimens without bevacizumab in patient tumor samples of NSCLC (74).

#### VEGF Induces the Differentiation of Macrophages From M1 to M2

TAMs promote angiogenesis by expressing a high level of VEGF. The lacked expression of costimulatory molecules on TAMs induces T cell tolerance and apoptosis. TAMs also promote immunosuppression in tumor by secreting cytokines that can suppress T cell recruitment and activation, such as IL-10, TGFβ, and prostaglandins (75). In addition to the recruitment of TAMs into tumor, VEGF signaling is also involved in the conversion of TAMs from the M1 to M2 phenotype. High levels of TAMs were observed in tumors with increased expression of stromal-cell-derived factor 1 alpha (SDF-1α), CXCL12, C-X-C motif chemokine receptor 4 (CXCR4) and VEGF in mouse tumor models (76, 77). Teresa E Peterson et al. have shown that dual inhibition of VEGFRs and Ang-2 reduced macrophage recruitment and promoted the polarization of TAMs to a M1 antitumor phenotype (78). Deng et al. also found that VEGF blockade potentiated antitumor efficacy in glioblastoma by reducing TAM recruitment into tumor (79), The combination of VEGFR and CXCR4 inhibitors also showed therapeutic effect in glioblastoma multiforme (GBM) xenografts (80).

#### VEGF Inhibits the Development and Activation of T Cells

T cells play an essential role in the antitumor immune response by directly killing tumor cells. Boosting the T cell immune response against cancer has become the primary goal of most immunotherapies. Low expression of VEGF was detected in T cells from tumor (81), suggesting that T cells might also promote angiogenesis. Ohm et al. found that the infusion of VEGF-A to tumor-bearing mice led to severe thymic atrophy resulted from a dramatic reduction in CD4^+^/CD8^+^ thymocytes (82). The inhibition of thymocyte maturation is mediated by the VEGFR2. These findings indicate that the VEGF signaling could directly inhibit T cell development. In addition, studies have shown that VEGF-A produced in the tumor microenvironment promotes T cell exhaustion by inducing the expression of co-inhibitory molecules in CD8^+^ T cell, and targeting VEGF-A/VEGFR signaling could reduce the expression of these suppressive genes (83).

VEGF-induced recruitment and expansion of suppressive immune cells in tumor inhibit the activation of tumor antigen-specific T cells. A lot of clinical and preclinical studies support that blockade of the VEGF/VEGFR signaling can enhance T cell response in tumor. Bevacizumab (Avastin) administration increased cytotoxic T cell levels in colorectal cancer and NSCLC patients (84, 85). Sunitinib treatment increase the levels of CD4^+^ and CD8^+^ T cell in mouse cancer models. Stronger cytotoxic activity and elevated expression of Th1 cytokine (Interferon-gamma, IFN-γ) were observed in these T cells from sunitinib-treated tumors (71). Similarly, Schmittnaegel et al. found that dual targeting of ANG2 and VEGFA increased the levels of effector CD8^+^ T cells in tumors (86). Furthermore, IFN-γ secreted by activated T cells has strong anti-angiogenic activity, suggesting that immunotherapy can also be antiangiogenic. The IFN-γR signaling could directly modulate the function and phenotype of vascular endothelial cells, and thereby normalize tumor blood vessels and promote effector T cell infiltration (87).

Lenvatinib is a RTK that specifically inhibits the kinase activities of VEGF receptors 1-3. Studies have shown that Lenvatinib reduced TAMs and increased the levels of effector CD8^+^ T cells. Combined with PD-1 blockade can further elevate the levels of activated CD8^+^ T cells, and thereby enhance antitumor immunity *via* the IFN signaling pathway (88).

### Synergism of Anti-Angiogenesis Inhibitors and ICB

Taken together, the VEGF signaling plays a pivotal role in the immunosuppressive TME which severely inhibits antitumor immune response. VEGF/VEGFR inhibition could reprogram the TME from immunosuppressive into immunostimulating by modulate the recruitment and function of immune suppressive cells and T cells. Therefore, anti-VEGF/VEGFR therapy not only has anti-angiogenic effects but also promotes immune response against cancer.

On the other hand, hypoxia-inducible factor 1-alpha (HIF-1α) up-regulates the expression of immune checkpoint molecules in tumor (83). VEGF-A directly increases the expression of PD-1 on activated CD8^+^ T cells and Tregs through VEGFR2 (83). Besides, elevated levels of IFN-γ in tumor resulted from VEGF signaling inhibition could induce the expression of PD-L1 on tumor cells. These mechanisms provide a theoretical basis for the combined treatment of advanced NSCLC with ICB and anti-angiogenic agents.

## Immunotherapy and Antiangiogenic Agents: Preclinical Study

Plenty of preclinical evidence also indicates that combining immunotherapy with anti-angiogenic inhibitors can improve the therapeutic efficacy in advanced NSCLC. It was reported that endostatin could improve the therapeutic effect of adoptive transfer of cytokine-induced killer cells (CIKs) for the treatment of lung carcinomas (89). Another preclinical study also showed that the VEGF inhibitor bevacizumab improved the effect of CIKs therapy in treating NSCLC (90). These findings provide evidence for the combination of anti-angiogenesis therapy and immunotherapy to treat lung cancer. In addition, the effects of different doses of antiangiogenic inhibitors on the combination with immunotherapy are also studied. A small dose of apatinib was enough to increase T cells infiltration, reduce hypoxia, and decrease the recruitment of TAMs into tumor (37, 91). Consistently, the combination of low-dose apatinib and PD-L1 antibody can significantly inhibit tumor growth and increase the survival time in mouse models (91).

## Immunotherapy and Antiangiogenic Agents: Clinical Data

Given that both the potential molecular mechanism and preclinical evidence support the combination of immunotherapy with anti-angiogenesis therapy, a number of clinical trials are underway to evaluate the safety and efficacy of this new therapy in NSCLC (**Table 1**). Preliminary data indicate that immunotherapy combined with anti-vascular therapy is a promising approach for the treatment of NSCLC.

**Table 1:** Clinical trials of the combination of anti-angiogenic inhibitors with immune checkpoint blockade in NSCLC.

Clinical trial	Patients	Targeted Agent	Primary Endpoint	Phase	Status
NCT01454102 (CheckMate 012)	Stage IIIB/IV NSCLC, first or subsequent line of therapy	Bevacizumab + nivolumab	SAE	I	Active, not recruiting
NCT02574078 (CheckMate 370)	Stage IV NSCLC	Bevacizumab + Nivolumab	PFS, OS	I/II	Completed
NCT02681549	Untreated brain metastases from melanoma or NSCLC	Bevacizumab + Pembrolizumab	BMRR	II	Recruiting
NCT02039674 (KEYNOTE- 021)	In participants with unresectable or metastatic NSCLC	Pembrolizumab + paclitaxel + bevacizumab	DLTs	I/II	Active, not recruiting
NCT02366143 (IMpower 150)	Stage IV non-squamous NSCLC	Atezolizumab + bevacizumab carboplatin + paclitaxel	PFS, OS	III	Completed
NCT02856425 (PEMBIB)	Solid tumors including NSCLC of adenocarcinoma and squamous	Nintedanib + Pembrolizumab	MTD of nintedanib, Safety	Ib	Recruiting
NCT02443324	LA/Unresectable/Metastatic NSCLC 0–3 prior lines of therapy	Ramucirumab + pembrolizumab	DLTs	I	Active, not recruiting
NCT02572687	LA/unresectable/metastatic/thoracic Malignancies	Ramucirumab + MEDI4736	DLTs	I	Completed
NCT02174172	Advanced or metastatic NSCLC	Bevacizumab + Atezolizumab	Dose of Atezolizumab	Ib	Completed
NCT03377023	Advanced or metastatic NSCLC	Ramucirumab + durvalumab	MTD, ORR	I/II	Recruiting
NCT03713944	Stage IV Non-squamous NSCLC	Bevacizumab + Atezolizumab	PFS, ORR	II	Active, not recruiting
NCT03647956	EGFR-mutant Metastatic NSCLC	Bevacizumab + Atezolizumab	ORR	II	Unknown
NCT03527108	Recurrent, Advanced, Metastatic NSCLC	Ramucirumab + Nivolumab	DCR	II	Recruiting
NCT03689855 (RamAtezo-1)	Stage IV, NSCLC, after progression on immune checkpoint blockers (ICBs)	Ramucirumab + Atezolizumab	ORR	I/II	Active, not recruiting
NCT03786692	Stage IV NSCLC in never smokers or possess a driver mutation	Bevacizumab + Atezolizumab	PFS	II	Recruiting
NCT03836066	LA/metastasis/high-intermediate tumor mutation burden in First Line NSCLC	Bevacizumab + Atezolizumab	PFS, OS	II	Recruiting
NCT03616691	LA/metastatic NSCLC after Failure with atezolizumab monotherapy	Bevacizumab + Atezolizumab	DCR	II	Not yet recruiting
NCT03786692	Stage IV NSCLC in never smokers or possess a driver mutation	Bevacizumab + Atezolizumab	PFS	II	Recruiting
NCT03735121	Previously Treated LA/Metastatic NSCLC	Bevacizumab + rHuPH20	Drug serum concentration	Ib/III	Recruiting

SAE, Serious Adverse Events; PFS, Progression-free survival; OS, Overall survival; BMRR, brain metastasis response rate; DLT, Dose-limiting Toxicity; MTD, Maximum Tolerated Dose ORR, Objective Response Rate; DCR, Disease control rate; LA, Locally Advanced.

### Nivolumab Combined With Bevacizumab

The combination between PD-1 blockade and bevacizumab was tested in the Checkmate012 phase I clinical trial (NCT01454102). Advanced NSCLC patients who failed in the first-line chemotherapy of platinum were divided into two groups, and treated with nivolumab or the combination of nivolumab with bevacizumab. The median PFS in the combination group was 37.1 weeks, while the nivolumab monotherapy group was 16 weeks in patients with squamous cancers and 21.4 weeks in patients with non-squamous cancers. Lower incidence of severe adverse events (AEs) (grade 3 and above) was observed in the combination. However, the objective response rates (ORR) are similar in these two groups. Follow-up studies are ongoing (12).

### Pembrolizumab Combined With Ramucirumab

The combination between ramucirumab and pembrolizumab has been studying by a multicenter phase I study (NCT02443324) in different types of cancers. 27 patients were recruited in this study. The objective reactions in these NSCLC patients were 30%. The median treatment time is 6.8 months or longer, and the median response time is 1.45 months. The most common serious AEs related to treatment in NSCLC patients were fatigue and myocardial infarction (7%) (92). The team has also expanded a multi-center, open-label Phase 1a/b trial to study ramoxiimab plus pembrolizumab in the treatment of advanced newly-treated NSCLC (N=26) (11). The results showed that 22 (84.6%) patients had any grade of treatment-related AEs, and hypertension is the most common side-effect (n = 4, 15.4%). The ORR of the treatment group was 42.3%. The ORR in patients with high PD-L1 expression levels (tumor proportion score (TPS)≥50%) and low levels (TPS 1%-49%) were 56.3% and 22.2%, respectively. The median PFS was 9.3 months in the treated group, and the patients with PD-L1 TPS 1%-49% were 4.2 months. The patients with PD-L1 TPS≥50% did not reach the median PFS. The median OS was not reached in the treated population.

### Atezolizumab Combined With Bevacizumab

The combination of bevacizumab with atezolizumab and chemotherapy was studied by IMpower150, which is a phase III randomized controlled clinical trial (NCT02366143). 1202 non-squamous NSCLC patients with stage IV or recurrent metastatic diseases who have not treated with chemotherapy were included. Patients were randomized 1:1:1 to receive atezolizumab combined with carboplatin + paclitaxel (ACP) (n = 402), atezolizumab combined with carboplatin + paclitaxel + bevacizumab (ABCP) (n = 400), carboplatin + Paclitaxel + Bevacizumab (BCP) (n = 400), after 4-6 courses of treatment, receive atezolizumab or bevacizumab or both for maintenance treatment until the disease progresses or no clinical benefit. The results of the study show that immunotherapy on the basis of the combination of bevacizumab and chemotherapy can prolong patient survival. The median PFS of the ABCP was 8.3 months, and the BCP was 6.8 months (HR: 0.59, P<0.0001). The median OS was 19.2 months for the ABCP group, and 14.7 months for the BCP group (HR: 0.78, P=0.02). The incidence of treatment-related serious AEs was 25.4% for ABCP group and 19.3% for BCP group. However, 77.4% of ABCP patients had grade 1-2 AEs. This study shows that, regardless of the PD-L1 expression, VEGFR or anaplastic lymphoma kinase mutation status, the use of ABCP can significantly improve PFS and OS in patients with metastatic non-squamous NSCLC (93). According to this study, the FDA approved the combination therapy of ABCP as the first-line treatment for metastatic non-squamous NSCLC in December 2018. This combination is currently being tested in hepatocellular carcinoma (HCC) as well. At the 2019 (ESMO) annual meeting, it was reported that atilizumab combined with bevacizumab and bisorafenib had better OS and PFS in patients with unresectable hepatocellular carcinoma (94).

### Apatinib Combined With SHR-1210

A single-arm phase II trial studying the combination of Apatinib with SHR-1210 was reported at the ASCO meeting in 2019. 96 patients were recruited in this study. Apatinib is a small TKI that primarily act on VEGFR-2, and SHR-1210 is another PD-1 antibody. These two drugs are developed in China. Patients failed at least one previous line of chemotherapy received intravenous infusion of SHR-1210 200 mg q2w combined with oral Apatinib 250 mg qd. The ORR of all evaluable patients was 30.8%. DCR was 82.4%. Median PFS was 5.9 months. The OS endpoint was not reached. Among the patients with bTMB 1.54 mutations/Mb, the ORR was 52.6%, and the DCR was 81.6%, suggesting that apatinib combined with SHR-1210 might have better therapeutic effect in patients with high tumor mutation burden (TMB) (95).

Overall, the combination of ICI and anti-angiogenic agents has shown encouraging results in treating advanced NSCLC. To achieve maximal therapeutic effect, a number of questions need to be addressed in future trails, including the effect of different anti-angiogenic inhibitors, the drug dose, the timing and schedule of the two type of drugs in the treatment etc.

## Conclusion

In this paper, we overviewed the updated knowledge of ICB, anti-angiogenesis, and the combination of these two kinds of therapies. A lot of preclinical studies have revealed the potential mechanisms of abnormal angiogenesis in the regulation of antitumor immunity in mouse tumor models, and support the application of combining immunotherapy and anti-angiogenesis for cancer treatment. The combination of immunotherapy and anti-angiogenesis is expected to enhance the efficacy of immunotherapy by converting the immunosuppressive TME to immunosupportive. Results of the ongoing clinical trials also support that the combination of ICB and anti-angiogenesis is a promising approach for the treatment of NSCLC. Translational studies and innovative clinical trials are needed in the future to address important questions not resolved in current studies, including the identification of biomarkers precisely the response to the combination therapy, optimizing the drug dose, administration schedule and the timing of the treatment.

## Author Contributions

JS and PZ conceptualized the idea for the review. SR and XX performed the literature search, analyzed cited references and wrote the first draft of the manuscript. HY, JS, and PZ wrote sections of the manuscript. All authors contributed to the article and approved the submitted version.

## Funding

This study is supported by grants from the National Key Research and Development Program of China (2016YFA0500304), the National Nature Science Foundation in China (NSFC) (81802853, 81773052, 81572806, 82002400), the Natural Science Foundation of Zhejiang Provincial (Y19H160116, Q18H160119), the Postdoctoral Science Foundation in China (2018M633237), the Guangzhou Science Technology and Innovation Commission (201607020038), the Science and technology projects of Guangdong Province (2016A020215086), the Guangdong Innovative and Entrepreneurial Research Team Program (2016ZT06S638), and the leading talents of Guangdong province program.

## Conflict of Interest

The authors declare that the research was conducted in the absence of any commercial or financial relationships that could be construed as a potential conflict of interest.

## Glossary

**Table:**

AE	Adverse event
ANG2	Angiopoietin-2
APC	Antigen-presenting cell
CCL2	C-C motif chemokine ligand 2
CCL20	C-C motif chemokine ligand 20
CCL22	C-C motif chemokine ligand 22
CCL28	C-C motif chemokine ligand 28
CIK	Cytokine-induced killer cell
CSF1	Colony stimulating factor 1
CTLA4	Cytotoxic T-lymphocyte-associated protein 4
CXCL12	C-X-C motif chemokine ligand 12
CXCR4	C-X-C motif chemokine receptor 4
DC	Dendritic cell
EC	Endothelial cell
EMA	Exponential moving average
FDA	Food and Drug Administration
FGFR	Fibroblast growth factor receptor
GBM	Glioblastoma multiforme
GM-CSF	Granulocyte-macrophage colony stimulating factor
HCC	Hepatocellular carcinoma
HIF-1α	Hypoxia-inducible factor 1-alpha
HPV	Human papillomavirus
ICAM1	Intercellular adhesion molecule 1
ICB	Immune checkpoint blockade
ICI	Immune checkpoint inhibitor
IDO	Indoleamine 2,3-dioxygenase
IFN-γ	Interferon-gamma
IL-10	Interleukin-10
IL-6	Interleukin-6
imDC	Immature dendritic cell
Jak2/STAT3	Janus kinase 2/Signal transducer and activator of transcription 3
MDSC	Myeloid-derived suppressive cell
MHC	Major histocompatibility complex
NF-κB	Nuclear factor κB
Nrp-1	Neuropilin 1
NSCLC	Non-small cell lung cancer
ORR	Objective response rate
OS	Overall survival
PD-1	Programmed cell death-1
PDGFR	Platelet-derived growth factor receptor
PD-L1	Programmed death ligand 1
PFS	Progress-free survival
PGE2	Prostaglandin E2
PI3K	Phosphoinositide-3-kinase
PIGF	Placental growth factor
PLCγ	Phospholipase C gamma
RTK	Receptor tyrosine kinase
SDF-1α	Stromal-cell-derived factor 1 alpha
TAM	Tumor associated macrophage
TGF-β	Transforming growth factor beta
TKI	Tyrosine kinase inhibitor
TMB	Tumor burden
TME	Tumor microenvironment
TPS	Tumor proportion score
Tregs	Regulatory T cells
VCAM1	Vascular cell adhesion protein 1
VEGF	Vascular endothelial growth factor
VEGFR	Vascular endothelial growth factor receptor
